# 1334. Radiographic Features Associated with Increased Microbiology Culture Yields in Pediatric Orbital Cellulitis

**DOI:** 10.1093/ofid/ofac492.1164

**Published:** 2022-12-15

**Authors:** Blake T Cirks, Kevin Claunch, Sarah Deperrior, Dan Adams

**Affiliations:** National Capital Consortium, Walter Reed National Military Medical Center, Bethesda, Maryland; Naval Medical Center Portsmouth, Portsmouth, Virginia; Naval Medical Center Portsmouth, Portsmouth, Virginia; Naval Medical Center Portsmouth, Portsmouth, Virginia

## Abstract

**Background:**

Orbital cellulitis is an invasive infection defined by its location posterior to the orbital septum and can result in severe clinical manifestations. Computed tomography (CT) is the recommended imaging modality to assess for extent of disease and the Chandler classification system is used. It has 5 stages (CS): I is preseptal; II has inflammation beyond the orbital septum; III has subperiosteal abscess; IV has orbital abscess; and V has cavernous sinus thrombosis. There is limited data in its association with microbiology culture results. As part of a larger study to capture epidemiological and microbiological trends in pediatric orbital we hypothesize that culture yields and identification of a causative pathogen would increase as CS increases.

**Methods:**

We performed a retrospective analysis using the US Military Health System database. ICD9/10 codes identified Individuals ≤ 21 years from June 2009 to September 2019 with orbital cellulitis. We excluded ophthalmia neonatorum and any cases without radiographic confirmation of orbital involvement. Demographic data, co-morbid diagnoses, imaging studies, microbiology results, and antibiotics prescribed were collected. CT reports were reviewed, and cases grouped by Chandler stage, (CS II-V). Descriptive statistics and χ ^2^ tests were used for categorical variables when appropriate.

**Results:**

130 cases met the inclusion criteria. 66.9% were male, the median age was 9 years old (IQR 4-17), and 55.4% had a prior diagnosis of sinusitis. 50.7% of cases were classified as CS II, 35.3% as CS III, 13.1% as CS IV, and 0.01% as CS V. Microbiology cultures were positive in 33.8% of cases. There was a significant difference between CS and any culture positivity with p = 0.011 and there was a significant trend between increasing CS and culture positivity with p < 0.003. *Staphylococcus aureus* and *Streptococcus intermedius* were the two most commonly identified pathogens, with no significant difference or trend associated with CS.

**Conclusion:**

Overall, our findings demonstrated microbiology yield being low but increases with radiographic characteristics and CS stage. Identification of causative pathogens could increase the likelihood for tailored definitive antimicrobial therapy in pediatric patients with orbital cellulitis.

**Disclosures:**

**All Authors**: No reported disclosures.

